# Genome-Wide Comparison of Cowpox Viruses Reveals a New Clade Related to Variola Virus

**DOI:** 10.1371/journal.pone.0079953

**Published:** 2013-12-03

**Authors:** Piotr Wojtek Dabrowski, Aleksandar Radonić, Andreas Kurth, Andreas Nitsche

**Affiliations:** Centre for Biological Threats and Special Pathogens, Robert Koch Institute, Berlin, Germany; Univ. of Texas HSC at San Antonio, United States of America

## Abstract

Zoonotic infections caused by several orthopoxviruses (OPV) like monkeypox virus or vaccinia virus have a significant impact on human health. In Europe, the number of diagnosed infections with cowpox viruses (CPXV) is increasing in animals as well as in humans. CPXV used to be enzootic in cattle; however, such infections were not being diagnosed over the last decades. Instead, individual cases of cowpox are being found in cats or exotic zoo animals that transmit the infection to humans. Both animals and humans reveal local exanthema on arms and legs or on the face. Although cowpox is generally regarded as a self-limiting disease, immunosuppressed patients can develop a lethal systemic disease resembling smallpox. To date, only limited information on the complex and, compared to other OPV, sparsely conserved CPXV genomes is available. Since CPXV displays the widest host range of all OPV known, it seems important to comprehend the genetic repertoire of CPXV which in turn may help elucidate specific mechanisms of CPXV pathogenesis and origin. Therefore, 22 genomes of independent CPXV strains from clinical cases, involving ten humans, four rats, two cats, two jaguarundis, one beaver, one elephant, one marah and one mongoose, were sequenced by using massive parallel pyrosequencing. The extensive phylogenetic analysis showed that the CPXV strains sequenced clearly cluster into several distinct clades, some of which are closely related to *Vaccinia viruses* while others represent different clades in a CPXV cluster. Particularly one CPXV clade is more closely related to *Camelpox virus*, *Taterapox virus* and *Variola virus* than to any other known OPV. These results support and extend recent data from other groups who postulate that CPXV does not form a monophyletic clade and should be divided into multiple lineages.

## Introduction

Variola virus (VARV) was one of the most lethal viruses known to humankind with an estimated total death toll of 300–500 million. Fortunately, owing to its narrow host range which only allowed infection of humans, VARV could be eradicated in a large-scale vaccination effort initiated by the WHO in 1967. The last known natural case of smallpox occurred in 1977 [1]. Based upon Edward Jenner’s discovery of the protective effect against VARV infection mediated by a poxvirus containing material taken from cows, which is generally assumed to contain cowpox virus (CPXV), live CPXV was considered to have been used initially as vaccine. During the vaccination campaign, however, the far less virulent Vaccinia virus (VACV) was used. However, the evolutionary relationship between VARV, VACV and CPXV is still unclear.

Because of a live vaccine’s potential to cause disease in immunosuppressed patients and in persons they are in close contact with, vaccination of the general public was halted around 1980 once the eradication of smallpox was accomplished [1]. While naturally occurring VARV infections are indeed no longer of concern – except in a bioterrorist context, ceasing to vaccinate has resulted in an increased number of human infections with zoonotic orthopoxviruses (OPV) such as monkeypox virus (MPXV) or CPXV [2]. The latter is of special interest for several reasons: (a) CPXV has the longest genome and the largest genetic repertoire of all known OPV [3,4], (b) CPXV has the broadest host range of all known OPV [5-7], (c) over the last years the German consultant laboratory for poxviruses has been diagnosing a rising number of CPXV infections of humans and animals [8-11] and (d) currently the monophyly of CPXV is being disputed [12,13]. Since only 12 full CPXV genomes have been published to date, we have sequenced the genomes of 22 additional CPXV strains collected from clinical cases mainly in Germany. Based on this data, we have performed extensive phylogenetic analyses that should contribute significantly to the on-going discussion. We also believe that the availability of further complete genomic data will enable a better understanding of the broad host range and the varying pathogenicity of CPXV.

## Materials and Methods

### Virus strain selection

All strains were collected as part of the routine diagnostic work at the German consultant laboratory for poxviruses. CPXV strains from independent cases involving ten humans, four rats, two cats, two jaguarundis, one beaver, one elephant, one marah and one banded mongoose were selected for sequencing. These strains were chosen so as to generate a genomic sequence of at least one strain from each infection event reported to the German consultant laboratory for poxviruses in the years from 2007 to 2009. All strains were obtained from German cases, except for one human case each from Lithuania and Austria. Information concerning the date and place of isolation, the host and public database accessions are given in Table S1, and a map showing the origin of the German strains is given in Figure S1.

### Virus culture and DNA isolation

All CPXV strains were propagated on Hep-2 cells; per 175 cm^2^ cell culture flask 2–3×10^7^ cells were infected at a multiplicity of infection of 0.2. At day 3 p.i. the cells from eight cell culture flasks were harvested, combined and washed with TSE (10 mM Tris HCl, 150 mM NaCl, 5 mM Na_2_EDTA, pH 8) buffer. The cells were incubated on ice for 10 min in 18 ml of TKE (10 mM Tris HCl, 10 mM KCl, 5 mM Na_2_EDTA, pH 8) buffer, followed by adding 2 ml of Triton X-100 and 50 µl of β-mercaptoethanol. After an additional 15 min of incubation on ice the nuclei were separated by centrifugation (1,500×g), the supernatant containing viral particles was ultra-centrifuged (20,000×g, 4°C, 30 min) and finally the pellet was resuspended in 800 µl of TKE buffer. DNA extraction was performed by using the Qiagen Genomic-tip 110/G and Qiagen Genomic DNA Buffer Set according to the manufacturer’s instructions for isolating genomic DNA.

### Sequencing

Between 500 ng and 1 µg of CPXV DNA, determined by measurement with the Qubit-dsDNA BR assay and instrument (Invitrogen), were used to generate libraries for 454 sequencing, utilizing the Library Kit for reads of 250 bases on average and the Rapid Library Kit for an average length of 450 bases. All libraries carried the sequences for amplification, the sequencing primer annealing and a key sequence as well as multiplex identifier sequences for multiplexing. The libraries were amplified by emPCR before sequencing. The emPCR step performed with the Roche chemistry amplifies a single molecule on a bead with capture oligos in a water-in-oil emulsion, so that monoclonal copies of the same fragment can be sequenced to generate a stronger signal than that from a single molecule. Sequencing was based on the 454 pyrosequencing chemistry by Roche, and two different kits were used: the standard chemistry to obtain a sequencing length of 250 bases and the XLR70 (or so-called titanium) chemistry. Base calling was performed by the instrument software, and further analyses were performed as described below.

### Sequence Assembly

After sequencing, the data obtained were mapped against the human genome sequence available at the National Center for Biotechnology Information (NCBI) by using the GS Mapper (Roche). Only non-mapping reads were used for further analysis. Assemblies were performed with the GS *de novo* Assembler (Roche) and Mimicking Intelligent Read Assembly (MIRA) [14]. For both assemblers, 20 assemblies were performed by using random permutations of the starting parameters. The assembly yielding the longest contigs from each of the assemblers was chosen for further processing. The Geneious software [15] was used to merge the contigs from the initial assemblies [16]. Finally, the original reads were mapped against these contigs to identify assembly errors.

### Alignment and Gene Extraction

For a comparison of all known genomic OPV sequences, including the ones newly generated in this project, an alignment was created by using the multiple alignment based on the fast Fourier transform (MAFFT) [17]. Furthermore, a Geneious plug-in was developed for the detailed examination of specific genes. This plug-in allows the user to select any number of genes annotated for a single species within a complex alignment. The plug-in then searches for matching open reading frames (ORF) in all other species within the alignment. A sub-alignment of all ORFs identified was extracted as a new document, and the ORFs were automatically annotated in order to allow easy identification of events such as truncation. The ORFs were also translated, and another new document was created containing the corresponding amino acid sequences. This facilitated the analysis of the effect of mutations on specific genes on both DNA and protein level and the quick identification of the genes most strongly affected.

### Phylogenetic Analysis

**Figure 1:**
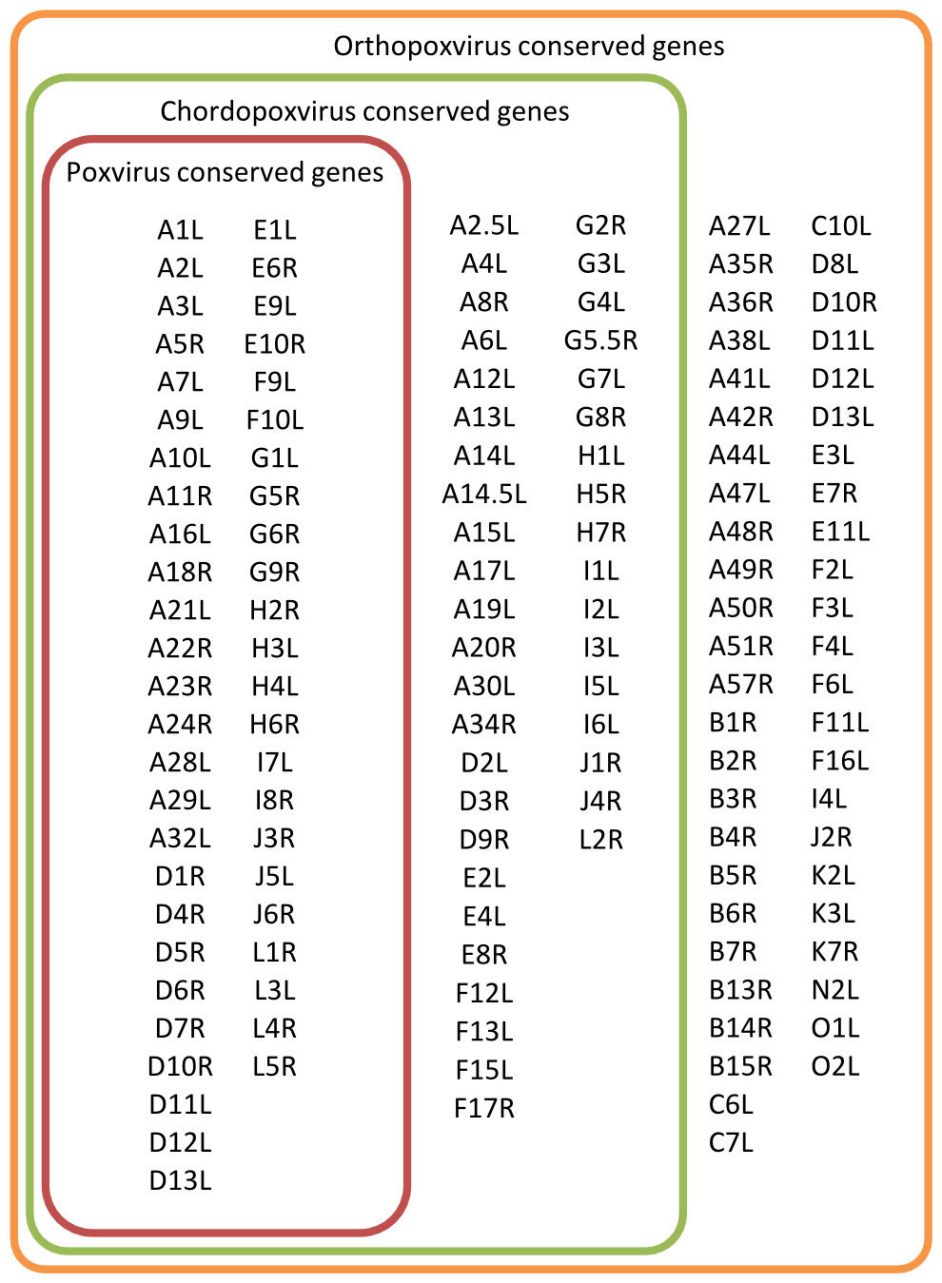
Groups of gene families that are specific for a phylogenetic level within the poxviruses. Genes are named after their VACV-Cop homolog. PVC: 49 genes; CVC: 49 PVC genes plus 41 CVC genes; OVC: 90 PVC/CVC genes plus 48 OVC genes.

In order to ensure the stability of the phylogenetic analysis, trees were calculated by using several different methods. First, the longest region containing sequence information for all taxa (VACV-Cop C12L – VACV-Cop B20R) was extracted from the genomic alignment, and a tree based on this was calculated by using an algorithm to estimate large phylogenies by maximum likelihood (PhyML) [18]. Then sub-alignments for specific sets of genes were extracted from the initial alignment by using the plug-in described previously. Both the resulting nucleotide and amino acid sequence alignments were then concatenated to create different pseudo-genome and pseudo-proteome alignments. The following gene sets were used: *Poxvirus* conserved genes (PVC, 49 genes on family level), *Chordopoxvirus* conserved genes (additional CVC; 41 genes on subfamily level) as described by Upton et al. [19] and a further set of genes with at least 80% conservation within all known OPV genomes (OVC; 48 genes) which we have identified by using a combination of BLAST and custom scripts. Additionally, two combinations of these sets were used: all genes conserved within either *Poxviruses* or *Chordopoxviruses* (PVC/CVC) and all genes conserved within either *Poxviruses*, *Chordopoxviruses* or *Orthopoxviruses* (PVC/CVC/OVC). Trees for the pseudo-genomes and pseudo-proteomes based on these three data sets were calculated by using PhyML for the nucleotide sequences and Neighbor Joining for the amino acid sequences. All genes used are listed with their VACV-Cop homologue’s ORF name in Figure 1. The parameters used in all tree DNA-based calculations were: Substitution model HKY85, estimated transition/transversion ratio, no invariable sites, 1 substitution rate category and NNI topology search. Branch supports were calculated by using Chi^2^ statistics. Since PhyML cannot handle stops in amino acid sequences, neighbor joining (Geneious 6.1.6) was used to calculate the amino acid-based tree. A 1000-fold bootstrap was performed and the support threshold was set to 95%.

Furthermore, we attempted to identify a small genomic region which could be used better than the customarily used HA gene to reconstruct the phylogenetic relationships suggested by the whole genome sequence-based tree. To this end we have calculated trees by using PhyML for each 1,000 bp sequence in the genome alignment and for each concatenation of two of these sequences. Each of these trees was then compared to the tree suggested by the whole genome sequence by using the Robinson Foulds metric [20]. This metric allows the calculation of a distance between two trees – the distance is defined by the number of operations which would be necessary to transform one of the trees into the other [20]. By this approach the sequence was identified that produced the tree most similar to the whole genome tree.

## Results

**Figure 2:**
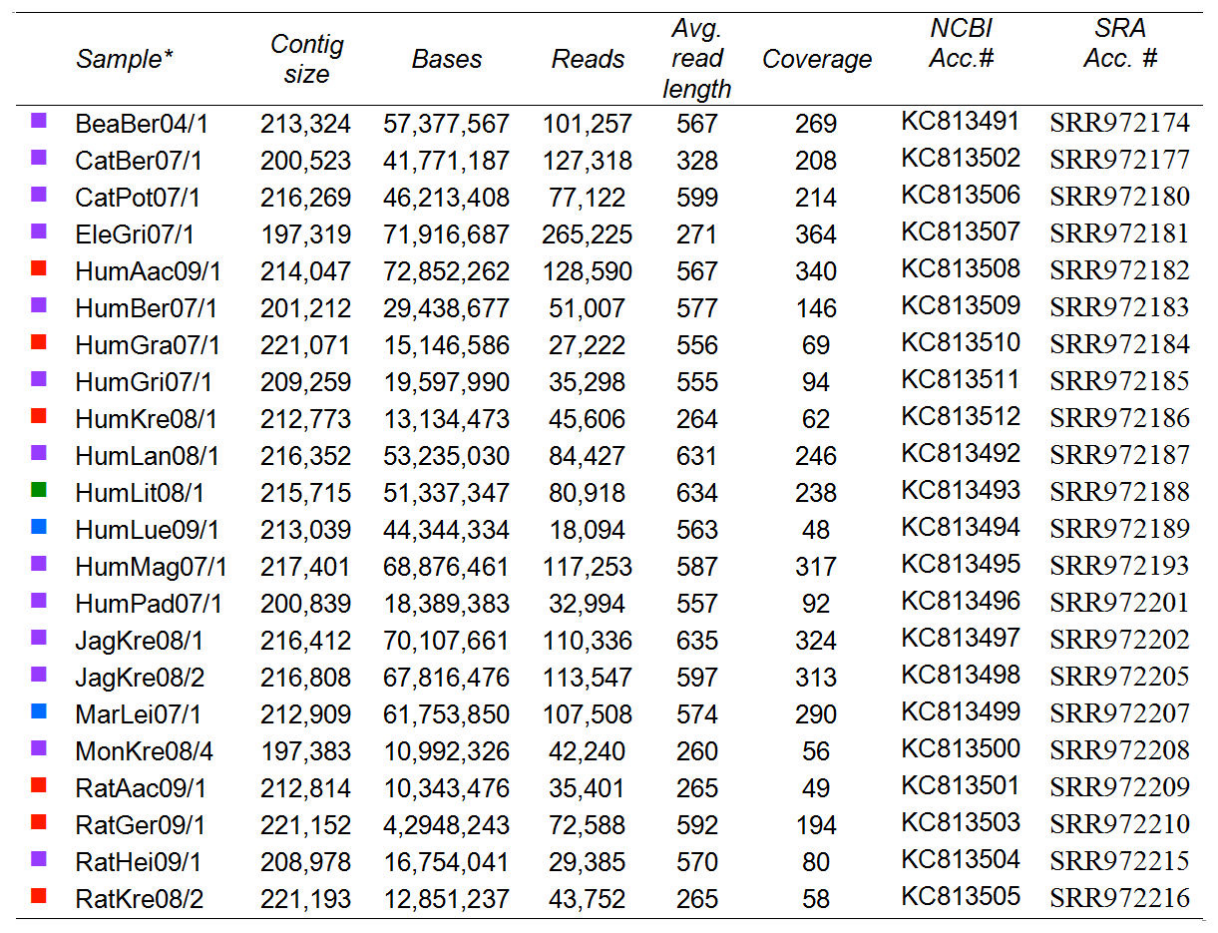
Overview of the sequencing results. *Nomenclature: the first three letters indicate the host species and the second three letters the geographical location/city from which the strain was obtained, followed by two digits for the year plus internal numbers. The color code on the left side of the table indicates the color of the corresponding clade in the tree figures.

Summarized information on the strains sequenced is given in Figure 2. In general, we were able to assemble all 22 CPXV genomes with a coverage ≥ 49× and to a contig length of ≥ 197 kb. All following analyses were based on these data. To our own data we added all genomic OPV sequences available from NCBI, including the sequences by Carroll et al. [13]. To establish a common ground with previous studies in this field, we selected the two sets of genes that were used by Upton et al. [19] and Gubser et al. [3] for phylogenetic analysis. The first set was conserved in all known *Poxviruses* and comprised 41 genes (PVC). The second set (CVC) was an additional collection of 49 genes that were conserved in *Chordopoxviruses*. Finally, we added a third set that comprised genes conserved throughout the OPV family (n = 48, OVC, Figure 1). Phylogenetic analysis was performed on each set and on combinations thereof, both on the nucleic acid and the amino acid level.

**Figure 3:**
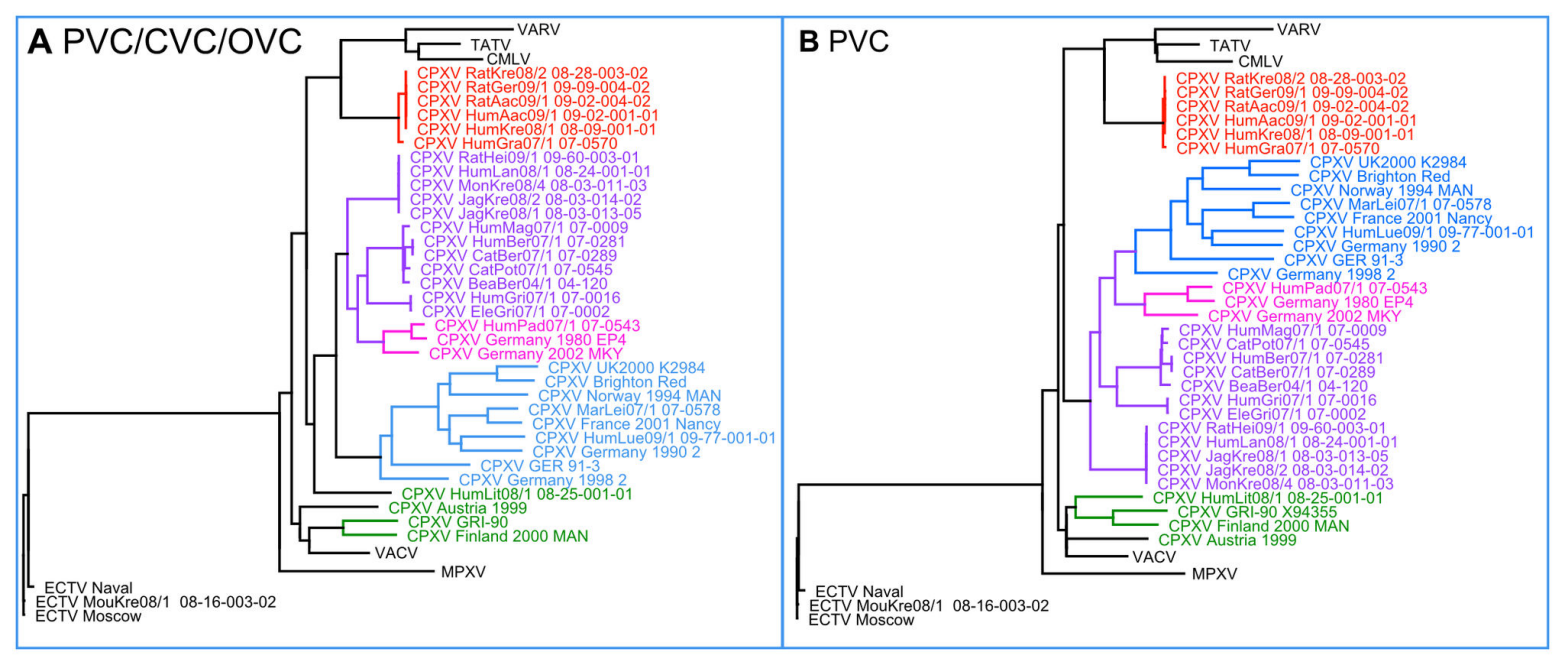
Phylogenetic trees resulting from the analysis of the DNA sequences of (a) *PVC/CVC/OVC* and (b) *PVC* gene families as shown in Figure 1. The VARV-like CPXV clade marked in red is visible when using any of the gene sets. VARV, VACV, CMLV and MPXV represent collapsed clades containing data from all whole genome sequences available in GenBank. All displayed branches have a Chi^2^-statistic value of at least 0.99.

Regardless of the gene set used in the analysis, most of the strains tended to cluster together. Based on this persistently resulting group formation across all trees, we defined several different groups of CPXV. These groups are highlighted in Figures 2 and 3. The purple and the red groups could be associated with specific patterns. In addition to others, the purple group encompasses all strains from an outbreak associated with feeder rats that caused several infections in different species between 2007 and 2009 [9], and the red group comprised strains mainly from Northern Germany between 2004 and 2009, some of which were associated with pet rats [11]. The other groups did not follow such simple attribution to the infectious source. In particular, the blue group contained strains from Germany, England, France and Norway that were spread over more than a decade. Nonetheless, these three major CPXV groups clearly formed monophyletic clades when calculating trees by using each gene set or a combination thereof (PVC, CVC, OVC, PVC/CVC and PVC/CVC/OVC – see Figure 3 and Figure S2). The only exception was the violet group which formed a paraphyletic instead of a monophyletic clade when using the PVC gene set. The small pink group, which contained sequences from three German cases between 1980 and 2002, was also clearly distinguishable from the others. In all analyses except that based on the PVC/CVC DNA, it was localized within the purple group, and we thus treated these three strains as belonging to the purple group. However, in order to highlight its different localization in the PCV/CVC DNA tree, we have colored it pink in our trees. Solely the four sequences marked in green (from Austria, Finland, Russia and Lithuania, 1990–2000) showed neither a clear clustering nor a consistent localization pattern within the tree. 

**Figure 4:**
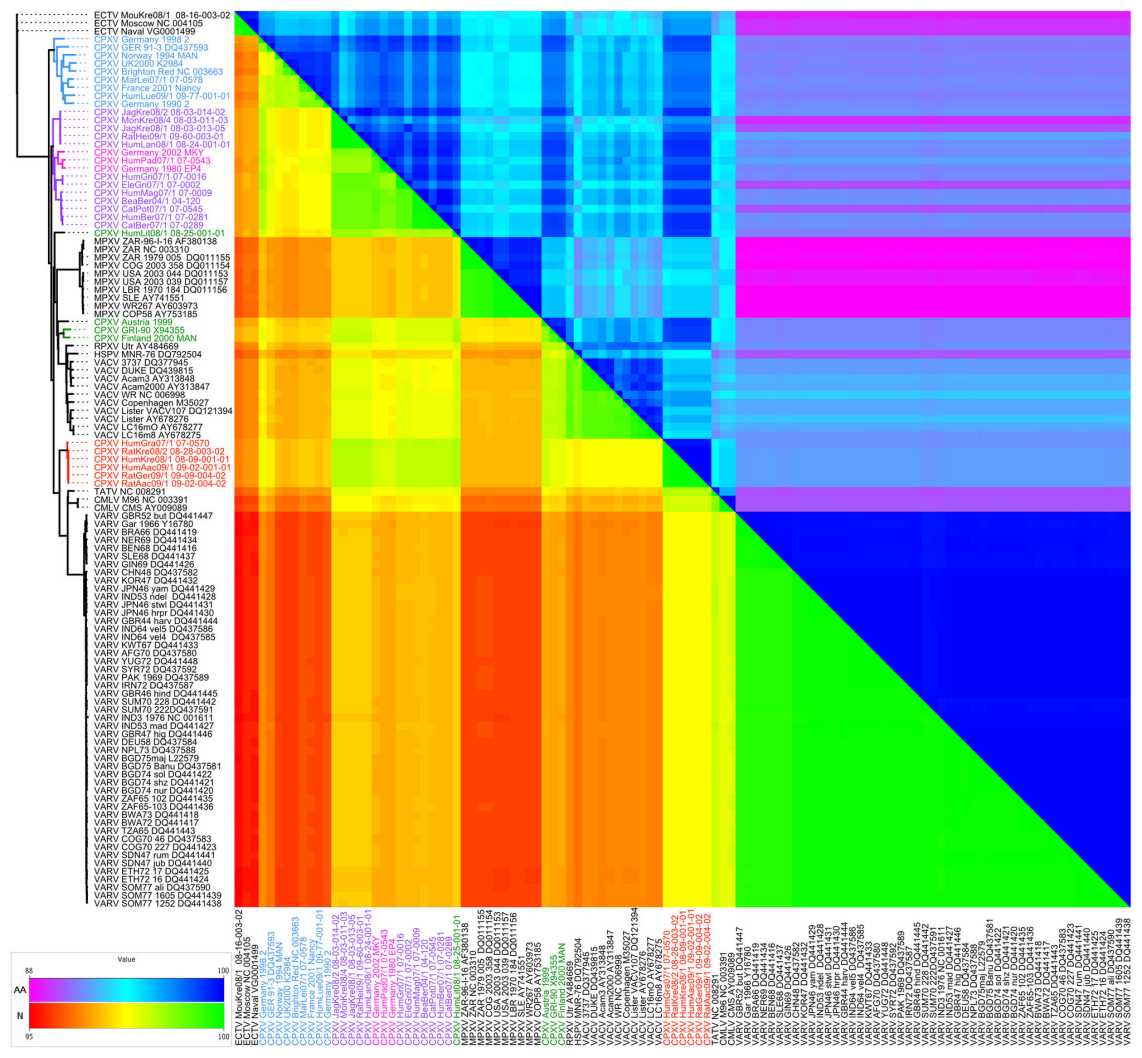
Identity between *Orthopoxviruses* based on the *Poxvirus*, *Chordopoxvirus* and *Orthopoxvirus* gene families. The lower left shows the identity on nucleic acid level, while the upper right shows the identity on amino acid level. The phylogenetic tree on the left is also based on a DNA sequence alignment of the *Poxvirus*, *Chordopoxvirus* and *Orthopoxvirus* gene families.

Based on the PVC/CVC/OVC gene set, the CPXV strains analyzed showed a degree of similarity towards sequences from the same group (97.8%–99.9% intra-group similarity) which was comparable to the degree of similarity seen in other OPV species (99.1%–99.9% intra-group similarity). However, the similarity between the different CPXV groups (lowest inter-group similarity: 97.1%, see Table 1) was significantly higher than the similarity one would expect within an OPV species. The clustering described previously [13] became evident already in the distance matrix of the alignment which is shown as a heatmap in Figure 4. 

**Table 1:** Minimum and maximum percentage of identical residues between sequences from different OPV clusters.

		CMLV	CPXV blue	CPXV green	CPXV red	CPXV violet	ECTV	MPXV	TATV	VACV	VARV
CMLV	min	99.9									
	max	100									
CPXV blue	min	96.4	97.8								
	max	97.2	100								
CPXV green	min	97.3	97.2	98.5							
	max	97.8	98.1	100							
CPXV red	min	98.1	97.1	98	99.9						
	max	98.2	97.9	98.5	100						
CPXV violet	min	97.4	97.5	97.9	98.1	98.6					
	max	97.7	98.6	98.7	98.5	100					
ECTV	min	95.9	96.4	96.8	96.6	96.7	99.7				
	max	96.2	97	97.2	96.8	97.1	100				
MPXV	min	96.4	96.7	97.4	97.1	97.1	96.2	99.5			
	max	96.5	97.2	97.6	97.1	97.3	96.5	100			
TATV	min	98.8	96.7	97.6	98.4	97.7	96.2	96.7	100		
	max	98.9	97.4	98	98.5	98	96.4	96.7	100		
VACV	min	97	96.9	98.1	97.7	97.6	96.5	97	97.3	99.1	
	max	97.2	97.7	98.5	97.8	98.1	96.7	97.2	97.4	100	
VARV	min	97.9	96	96.9	97.7	96.9	95.5	96	98.2	96.6	99.6
	max	98.1	96.7	97.3	97.8	97.3	95.8	96.2	98.3	96.7	100

Alignments of DNA sequences of the genes from the *PVC/CVC/OVC* gene families as shown in Figure 1 were used for the calculation.

A condensed presentation is given in Table 1 that contains the identity between the different OPV groups on the nucleotide level. Interestingly, the distances between the CPXV groups (97.1%–98.7%) were similar to the distance between the red CPXV group and Taterapox virus (TATV) (98.4%–98.5%), indicating the heterogeneity among the CPXV. Also, the red CPXV group was nearly as similar to VARV (97.7%–97.8%) as to TATV (98.2%–98.3%), which was a significantly higher degree of identity than that observed for monkeypox virus (MPXV) (96%–96.2%) and VACV (96.6%–96.2%). Both the blue and the violet CPXV group were less similar to VARV (96%-96.7% and 96.9%–97.3%, respectively) than to *Taterapox virus* TATV (98.2%–98.3%).

**Figure 5:**
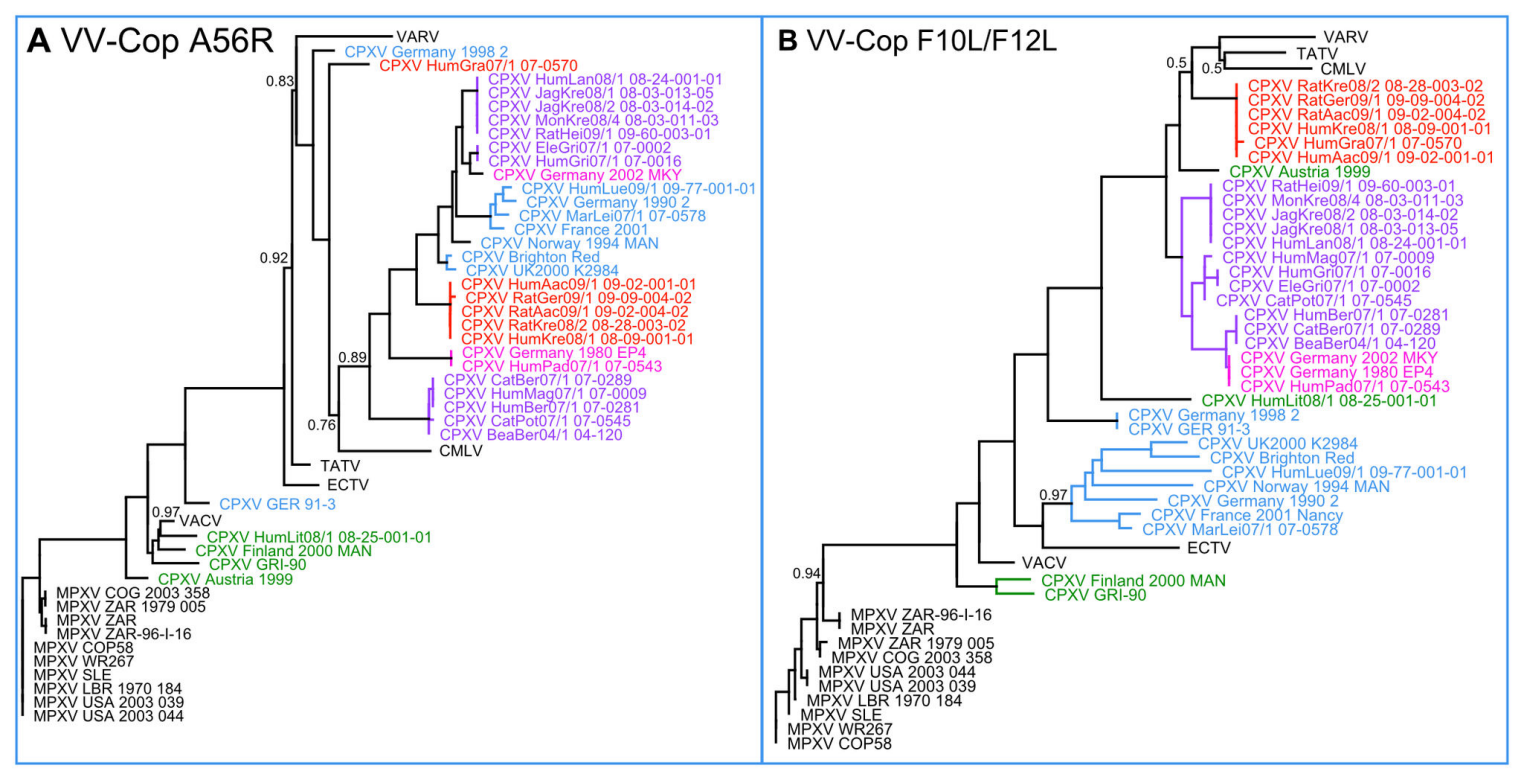
Phylogenetic trees resulting from the analysis of the DNA sequences of (a) the HA gene and (b) 1 kb from VACV-Cop F10L and VACV-Cop F12L each. The different CPXV clades cannot be resolved when using the sequence of the HA gene for phylogenetic analysis, as they all cluster together. In contrast, the concatenated sequences from F10L and F12L can be used to achieve a clear separation of all groups visible in Figure 3a. VARV, VACV, CMLV and ECTV represent collapsed clades containing data from all whole genome sequences available in GenBank. All displayed branches, unless otherwise indicated, have a Ch^i2^-statistic value of at least 0.99.

A particularly interesting aspect of this pronounced separated clustering was the localization of the newly identified red group. Regardless of which gene set was used for the analysis, this red group always localized significantly closer to the *Camelpox virus* (CMLV), TATV and VARV clades than did any other CPXV group, including other CPXV strains isolated from humans. Interestingly, this closer relationship to VARV had not been detected previously during routine diagnostic work. This is due to the fact that the sequence most widely used for phylogenetic analysis in diagnostics was that of the HA gene (VACV-Cop A56R) [21]. As can be seen in Figure 5a, a phylogenetic tree based upon the HA sequence is unable to separate the groups into different clades which are clearly visible when using the whole genome sequences for any of the applied gene families, thus hiding the diversity exhibited by CPXV. 

This led us to search for a part of genomic sequence which performed better under this aspect. Phylogenetic trees were automatically calculated from up to two short sequence stretches of the genome, and the Robertson Foulds distance was calculated between these trees and the tree based on the sequences of *Poxvirus*, *Chordopoxvirus* and *Orthopoxvirus* gene families (Figure 3a). We have thus identified a combination of 1,000 bp from the VACV-Cop genes F10L (VACV-Cop: bases 40253–41245) and F12L (VACV-Cop: bases 42635–43630) each (Robinson Foulds distance: 116 operations, Figure 5b) as the combination suited best for approximation of the whole genome tree. As can be seen in Figure 5, all of the previously described groups of CPXV – with the exception of the green group which does not show a consistent clustering in the full genome analyses based on the different gene sets – could be clearly separated through phylogenetic analysis using this sequence. Also the higher similarity of the red CPXV group to VARV is visible. For perspective, the gene performing worst was the VACV-Cop gene E11L (Robinson Foulds distance: 208 operations) while the standard HA gene (VACV-Cop gene A56R) occupied the middle ground with a Robinson Foulds distance of 154 operations (Figure 5a). 

## Discussion

The examination of complete genomes provides more detailed insight into phylogenetic relations of pathogens compared to previous studies that usually had been restricted to the analysis of short genomic regions. In the present study, we analyzed phylogenetic relations of OPV by adding the full genome sequences of 22 newly isolated CPXV strains to the 93 OPV full genome sequences that became publicly available recently, 12 sequences of which were CPXV.

The results of our phylogenetic studies including the new 22 genomic sequences of CPXV strains isolated from clinical infections in Europe underline the results of previous studies that argued that CPXV was not a monophylum [12,13]. Our analysis of the nucleotide sequences of gene families specific for *Poxviruses* (PVC), *Chordopoxviruses* (CVC) and *Orthopoxviruses* (OVC) suggests that in reality the so-called taxon CPXV is paraphyletic. Recently, Carroll et al. identified two CPXV clades which were defined as one CPXV-like clade and one VACV-like clade [13]. With the additional 22 strains sequenced in this study we were able get a more detailed concept of these clades. To our surprise we found a new clade of CPXV, consisting of strains that were involved in cases in which feeder rats transmitted a CPXV infection to humans and other animals [9], that is genetically more closely related to CMLV, TATV and VARV than any other CPXV. The other strains which seem to occur naturally in Germany [10] fell into the previously described CPXV-like clade. Figure S3 shows an unrooted phylogenetic tree that clearly visualizes the relationship. These results suggest that the CPXV-like clade is not monophyletic and can be separated into two different clades.

The *Orthopoxviruses* CMLV, TATV and VARV are known to have a narrow host range, while CPXV display a broad host range. A general observation is that OPV with a broad host range cause mild infections in the respective hosts, while OPV with a narrow host range often cause severe to lethal infections in their hosts [2]. Although it would be daring to speculate that the strains in this new clade show a narrowed host range or a higher pathogenicity in their hosts, it is striking that an infection of the pet rats with these CPXV strains often had a lethal outcome. This observation was made regarding a number of pet rats that transmitted the virus to humans during the years 2008 and 2009 in Germany, as already reported [11]. For one strain in this clade isolated from a human in Graz, Austria in 2007 no additional data is available. It remains unclear if this strain might also have been associated with a pet (rat) or was transmitted via a different, maybe more common infection route like contact to cats. Every phylogenetic analysis we performed by using different approaches showed that this strain was not related to another Austrian strain from 1999, which suggests that there is no geographical correlation. However, it is appropriate that all the strains that can be attributed to the same source of infection, as was observed for the feeder rats or the pet rats, clustered together in one clade. 

The previously defined VACV-like group was a monophyletic clade containing VACV and the three CPXV strains GRI-90, FIN2000-MAN and AUS1999_867 [13]. This is in agreement with our results. However, in our study we could not identify additional strains that would fall into this clade. Naturally occurring strains that would fall into the VACV-like clade were not being detected in Germany or Central Europe over the last few years. The only strain that could be related with this group in one phylogenetic analysis was obtained from a human CPXV case in Lithuania that seemed to switch its location in the phylogenetic tree, depending on the genes analyzed. This strain clustered to VACV only when the OVC were considered in the phylogenetic analysis. The identification of more sequences belonging to this clade might help assess if some of the so-called VACV-like CPXV are a monophyletic clade within the VACV clade. With the data available at present, it is not possible to draw final conclusions on whether or not some CPVX strains belong to the VACV clade. 

Our results do, however, show clearly that CPXV does not form a monophyletic clade within the Orthopoxvirus phylogeny. This strengthens the division into a VACV-like and a CPXV-like clade as presented by Carroll et al. [13]. In addition, we have identified a third, clearly separate clade which clusters closer to VARV in our phylogenetic analyses than any previously described CPXV.

As more genomes of OPV will be sequenced, the phylogenetic picture will get sharper and we will finally better understand the development of VACV and even VARV. The better knowledge of the relations of CPXV to VACV and VARV may also help elucidate the origin of these OPV. The possibility to generate and analyses genome sequences of OPV from historical clinical preparations would be of great benefit.

While this work focused on the phylogenetic analysis of the newly generated genome sequences, the data presented here will hopefully facilitate further investigation into the makeup and function of OPV genomes, such as the effect of the presence of specific genes on the host range or on virulence.

Most of the phylogenetic results we obtained were only evident if sets of genes instead of single genes were analyzed. Particularly, the HA sequence often used for phylogenetic analysis was not mirroring the results obtained by whole genome analyses. Assuming that the analysis of the whole genome reveals the best approximation to reality, we postulate that the phylogenetic relations derived from HA gene sequencing are not as reliable as assumed previously. Since it is not clear how soon whole genome sequencing will become available to most of the laboratories, and because bioinformatics analysis of this kind of data is challenging, we identified two additional gene regions which might be included in future phylogenetics of OPV: F10L and even better in combination with F12L. Identification of further strains and phylogenetic analyses based on these two genes in combination with whole genome sequencing will be necessary to assess the future use of these surrogate gene regions.

## Supporting Information

Figure S1 **Map of Germany with red dots indicating place of origin of sequenced strains.** The shades of blue indicate the number of strains acquired from each state. Two strains from outside of Germany (one from Graz, Austria and one from Vilnius, Lithuania) are not shown on this map.
(TIFF)

Figure S2 **Phylogenetic trees resulting from the analysis of the DNA sequences of (**a**) *Chordopoxvirus*, (**b**) *Orthopoxvirus*, and (**c**) *Poxvirus* and *Chordopoxvirus* and (**d**) the amino acid sequence of *Poxvirus*, *Chordopoxvirus* and *Orthopoxvirus* gene families as shown in Figure 1.** Again, the VARV-like CPXV clade marked in red is visible when using any of the gene sets. VARV, VACV, CMLV and MPXV represent collapsed clades containing data from all whole genome sequences available in GenBank. All displayed branches on the DNA sequence-based trees, unless otherwise indicated, have a Chi^2^-statistic value of at least 0.99. All branches on the amino acid sequence-based tree (1000-fold bootstrap, support threshold 95%) have a 100% consensus support unless otherwise indicated.
(TIFF)

Figure S3 **Phylogenetic tree resulting from the analysis of *Poxvirus*, *Chordopoxvirus* and *Orthopoxvirus* gene families as shown in Figure 1, displayed as unrooted tree with no branch transformation.** The VARV-like CPXV clade is marked in red and is clearly closer to TATV, CMLV and VARV than are any other CPXV, and it represents a clearly distinct clade.
(TIFF)

Table S1 **Table of additional data for the sequenced strains.**
(XLSX)
