# Supplementary figures and images for: Genome-Wide Comparison of Cowpox Viruses Reveals a New Clade Related to Variola Virus
Source: PLoS One. 2013 Dec 3;8(12):e79953. doi: 10.1371/journal.pone.0079953 (PMC3848979; doi:10.1371/journal.pone.0079953)

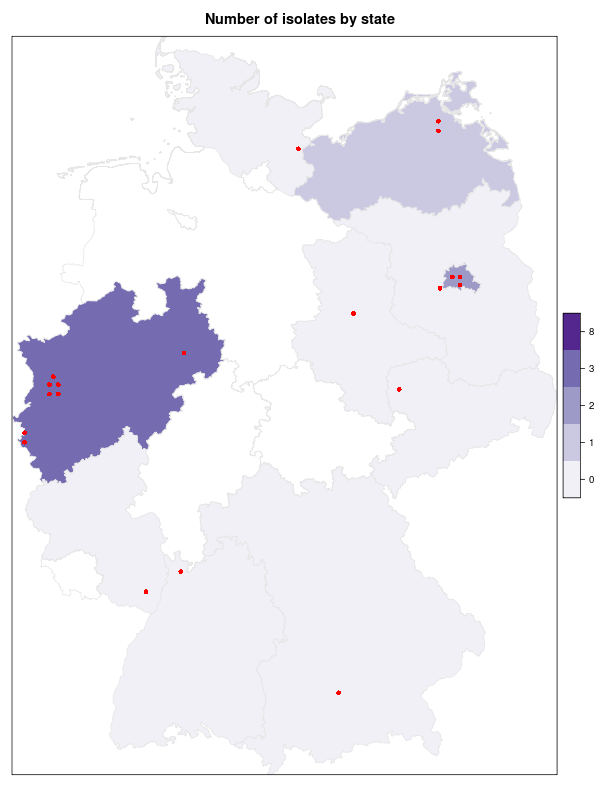

Supplement: Figure S1 — Map of Germany with red dots indicating place of origin of sequenced strains. The shades of blue indicate the number of strains acquired from each state. Two strains from outside of Germany (one from Graz, Austria and one from Vilnius, Lithuania) are not shown on this map. (TIFF) [file pone.0079953.s001.tiff]

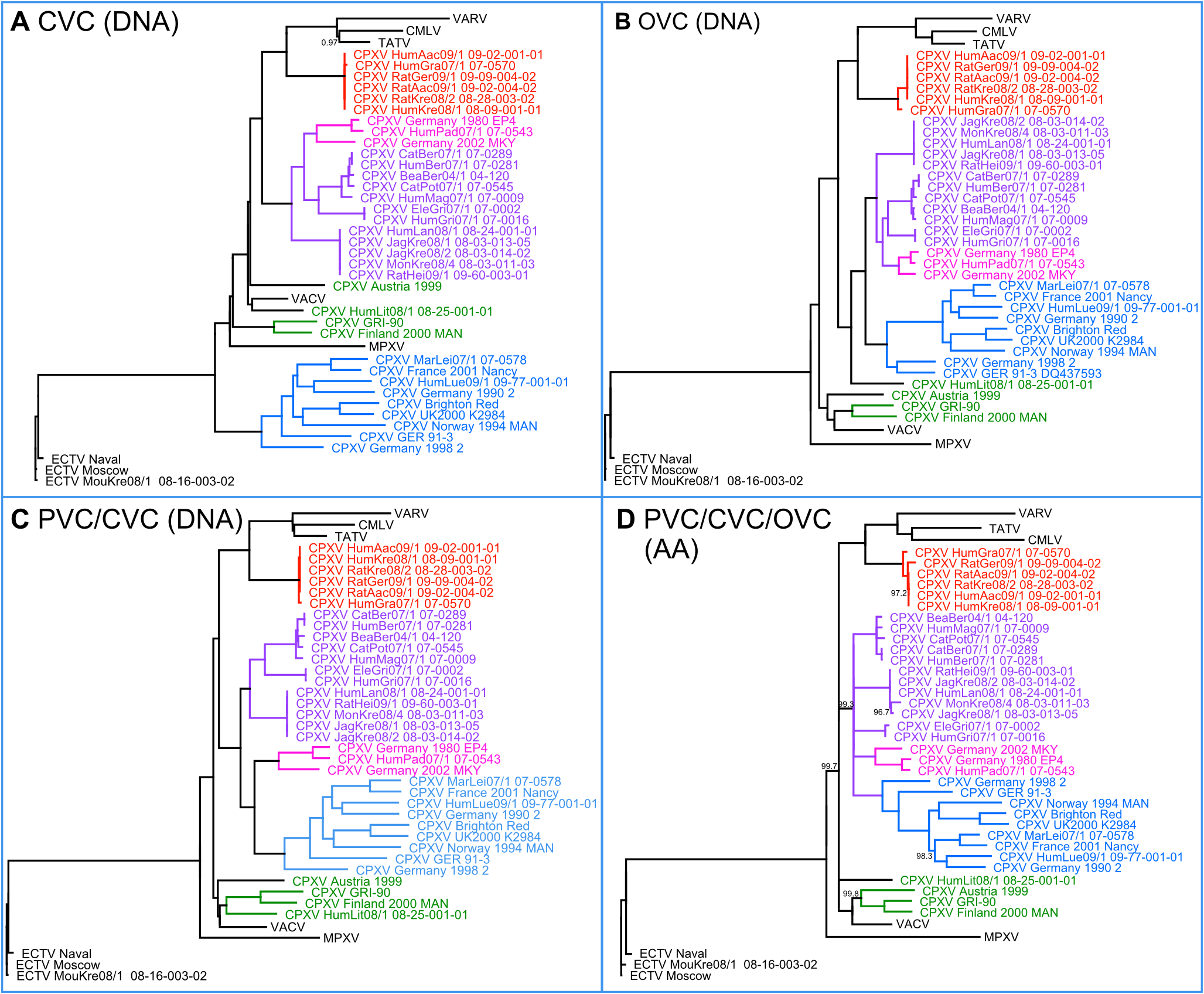

Supplement: Figure S2 — Phylogenetic trees resulting from the analysis of the DNA sequences of (a) Chordopoxvirus, (b) Orthopoxvirus, and (c) Poxvirus and Chordopoxvirus and (d) the amino acid sequence of Poxvirus, Chordopoxvirus and Orthopoxvirus gene families as shown in Figure 1. Again, the VARV-like CPXV clade marked in red is visible when using any of the gene sets. VARV, VACV, CMLV and MPXV represent collapsed clades containing data from all whole genome sequences available in GenBank. All displayed branches on the DNA sequence-based trees, unless otherwise indicated, have a Chi2-statistic value of at least 0.99. All branches on the amino acid sequence-based tree (1000-fold bootstrap, support threshold 95%) have a 100% consensus support unless otherwise indicated. (TIFF) [file pone.0079953.s002.tiff]

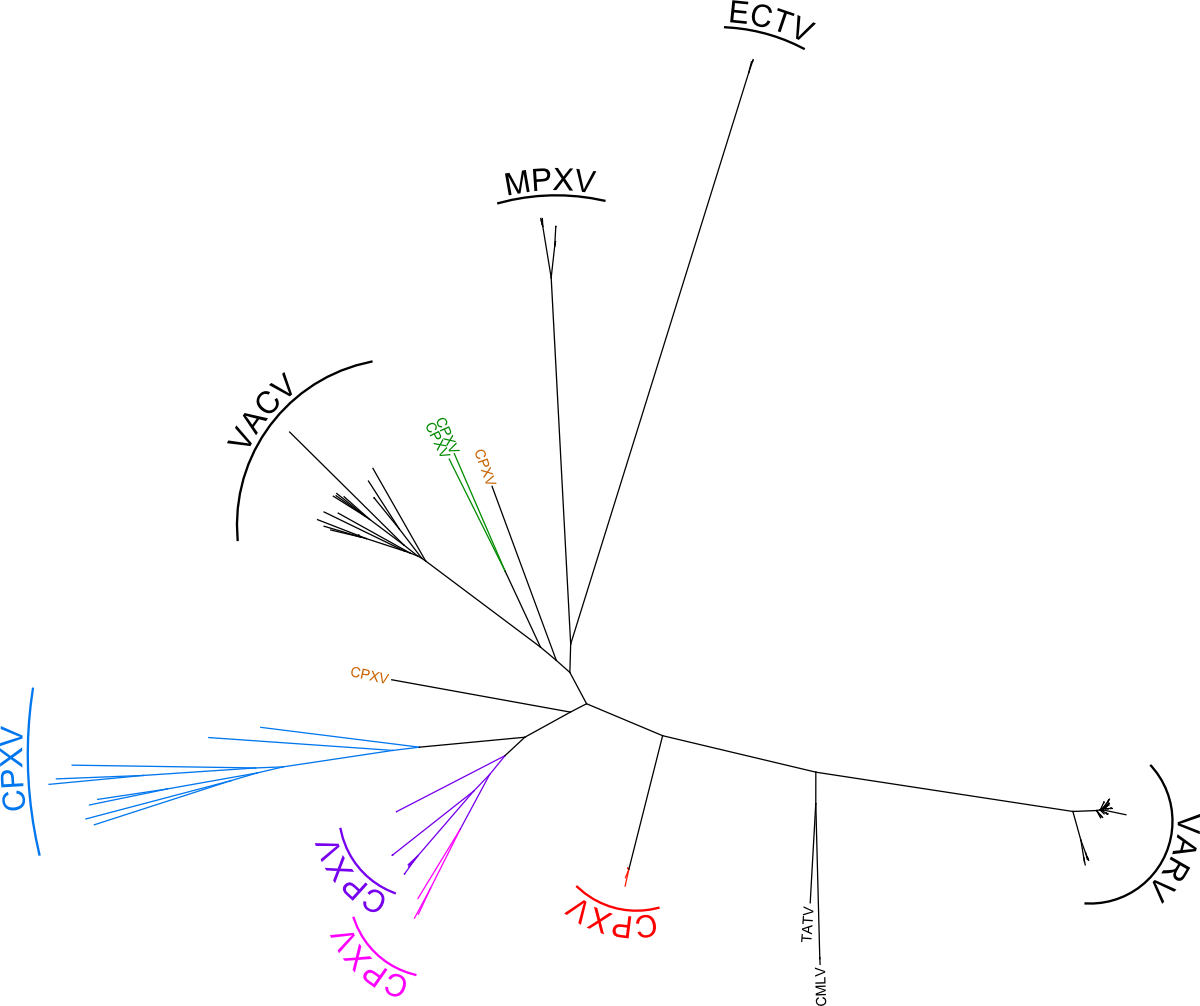

Supplement: Figure S3 — Phylogenetic tree resulting from the analysis of Poxvirus, Chordopoxvirus and Orthopoxvirus gene families as shown in Figure 1, displayed as unrooted tree with no branch transformation. The VARV-like CPXV clade is marked in red and is clearly closer to TATV, CMLV and VARV than are any other CPXV, and it represents a clearly distinct clade. (TIFF) [file pone.0079953.s003.tiff]
